# Skeletal posterior crossbite in patient with mandibular asymmetry: an alternative solution

**DOI:** 10.1590/2177-6709.26.3.e21bbo3

**Published:** 2021-06-25

**Authors:** Fábio Lourenço ROMANO, Marcelo Antônio MESTRINER

**Affiliations:** 1 Universidade de São Paulo, Faculdade de Odontologia de Ribeirão Preto, Departamento de Clínica Infantil, área de Ortodontia (Ribeirão Preto/SP, Brasil).; 2 Private practice (Ribeirão Preto/SP, Brasil).

**Keywords:** Facial asymmetry, Palatal expansion technique, Orthognathic surgery, Corrective Orthodontics

## Abstract

**Introduction:**

Skeletal posterior crossbite (SPCB) has a multifactorial etiology, as it may be caused by parafunctional habits, atypical position of the tongue, tooth losses and maxillary or mandibular transverse skeletal asymmetries. Skeletal involvement may lead to facial changes and an unfavorable aesthetic appearance. The treatment of SPCB diagnosed in an adult patient should be correctly approached after the identification of its etiologic factor. Surgically-assisted rapid maxillary expansion (SARME), one of the techniques used to correct SPCB in skeletally mature individuals, is an efficient and stable procedure for the correction of transverse discrepancies that may be performed in the office or in a hospital.

**Objective:**

This study discusses the results of asymmetrical SARME used to correct unilateral SPCB associated with transverse mandibular asymmetry.

**Conclusion:**

The treatment alternative used in the reported case was quite effective. At the end of the treatment, the patient presented adequate occlusion and facial aesthetics.

## RESUMO

**Introdução:**

A mordida cruzada posterior esquelética (MCPE) apresenta etiologia multifatorial, podendo ser causada por hábitos parafuncionais, posição atípica da língua, perdas dentárias e assimetrias esqueléticas transversais da maxila ou da mandíbula. Alterações faciais podem estar presentes quando há envolvimento esquelético, levando a estética desfavorável. O tratamento da MCPE, quando diagnosticada no paciente adulto, requer abordagem correta, com identificação do fator etiológico. Entre as técnicas utilizadas para correção da MCPE em pacientes esqueleticamente maduros, cita-se, em especial, a Expansão Rápida de Maxila Assistida Cirurgicamente (ERMAC). Essa modalidade tem se mostrado bastante eficiente na correção dos problemas transversais, apresenta estabilidade e pode ser realizada em ambiente ambulatorial ou hospitalar.

**Objetivo:**

O objetivo do presente trabalho será discutir os resultados da ERMAC assimétrica para correção da MCPE unilateral associada a assimetria transversal da mandíbula.

**Conclusão:**

A alternativa de tratamento utilizada no caso relatado mostrou-se bastante eficiente. Ao fim do tratamento, o paciente apresentou adequada oclusão e boa estética facial.

## INTRODUCTION

Adults have been increasingly seeking orthodontic treatment. Some patients have skeletal and facial asymmetries in addition to occlusal problems, which may worsen their condition or complicate their treatment. The human face is not perfectly symmetrical, but facial asymmetries are so small in most cases that they are hardly noticed in social life.^1^ However, differences between sides of the face in patients with skeletal asymmetries of the maxillary bones may be visible and, therefore, disturbing and uncomfortable. Facial asymmetries smaller than 3 to 4 mm usually go unnoticed by the layperson. Orthodontists, in contrast, may see asymmetries as small as 2 mm.^2^ Mandibular shift and asymmetries are more visible^1^ and are usually associated with congenital malformation or deformity of the craniofacial skeletal structures, with asymmetrical growth or with mandibular posture compensation.^1^ These factors may be the origin of unilateral skeletal posterior crossbite (SPCB). This type of malocclusion rarely has a spontaneous resolution, and requires a specific diagnosis to detect the skeletal and dental components involved. Intervention time is also a decisive factor in the treatment of SPCB^3^ ^,^ ^4^. In children and young adolescents, conventional rapid maxillary expansion (RME) using expanders is an efficient method to correct SPCB.^5^ ^,^ ^6^ ^,^ ^7^ However, when used for older adolescents and adults, dentoalveolar effects are predominant, with little or no skeletal expansion.^7^ This may lead to root resorption of the teeth used for anchorage, excessive dental tipping, dehiscence, fenestration and expansion failure.^8^ ^,^ ^9^ For these patients, other treatment options, such as miniscrew-assisted rapid palatal expansion (MARPE) and surgically-assisted rapid maxillary expansion (SARME) should be considered.^10^ ^,^ ^11^ Treatments using either of these techniques have positive and stable results.^10^ ^-^ ^15^ SARME consists of a bilateral Le Fort osteotomy and separation of the midline at the incisor region.^13^ ^,^ ^14^ It may be performed in the office, under local anesthesia, or in the hospital, when it requires general anesthesia.^15^The technique may be adapted to correct individual needs and include, for example, pterygomaxillary disjunction to ensure greater posterior expansion and unilateral osteotomy to decrease the areas of resistance and promote asymmetrical expansion.^15^ ^-^ ^19^ When SPCB is unilateral and a result of mandibular asymmetry, sagittal split ramus osteotomy (SSRO) is an option. However, this complex and invasive technique has high risks and may trigger undesirable side effects.^20^ In cases of unilateral SPCB, expansion is not enough to completely correct malocclusion. Most cases will also need further orthodontic treatment to correct the anteroposterior and vertical position of teeth and achieve normal occlusion.^4^ ^,^ ^21^

Thus, the present study discusses the results of asymmetrical SARME used to correct unilateral SPCB associated with transverse mandibular asymmetry, and presents the case of an adult woman with Class II, division 2, left subdivision malocclusion and unilateral SPCB. 

## CASE REPORT

### DIAGNOSIS AND DESCRIPTION

A 45-year-old woman presented with a complaint that she described as: *“I’m biting with my teeth in an inverted position in the posterior region”.* Her general health was good and she did not report any significant medical problem. She had good gingival health, but defective restorations. 

**Figure 1:**
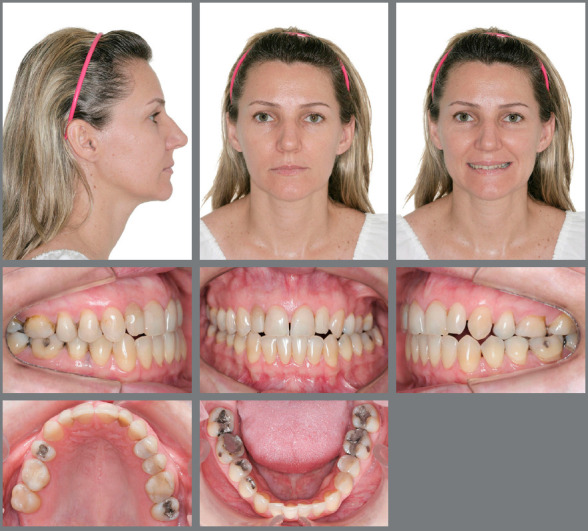
Initial facial and intraoral photographs.

She had a slightly concave profile and well-proportioned facial thirds. Her face was slightly asymmetric, as the left side of the mandible seemed to be larger than the right side. Lip seal was passive, her smile was asymmetric, and her left buccal corridor was larger than the right one (Fig 1). 

She had Class II malocclusion, division 2, subdivision left because of loss of tooth #25 and consequent mesial movement of teeth #26 and #27, together with a reduced axial inclination of her maxillary incisors. Examinations revealed overbite, an edge-to-edge relationship and maxillary lateral incisors with a reduced mesiodistal diameter. 

**Figure 2:**
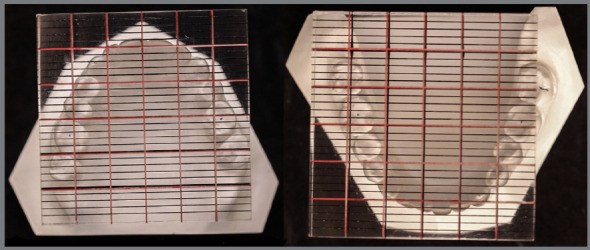
Analysis of dental arch symmetry, using a measuring plate (Schmuth).

The mandibular midline was slightly deviated to the right of the facial midline, and the maxillary, to the left. Left unilateral SPCB and slightly expanded maxillary teeth in the left side were not enough to avoid the crossbite. Occlusal wear facets were found mainly in the anterior teeth, because of malocclusion (Fig 1). Analyses using plaster models revealed asymmetries in the maxillary and mandibular arches (Fig 2 and Table 1). 

**Table 1:** Analysis of dental arch symmetry.

Anteroposterior	Teeth	Arches	
		Maxillary	Mandibular
	Canines	#13: 1 mm mesial to #23	#33: 1 mm mesial to #43
	Molars	#26: 3 mm mesial to #16	#36: 1 mm mesial to #46
Transverse	Teeth	Arches	
		Maxillary	Mandibular
	Canines	#23: 2 mm expanded to #13	#33: 6 mm expanded to #43
	Molars	#26: symmetric to #16	#36: 8 mm buccal to #46

**Figure 3:**
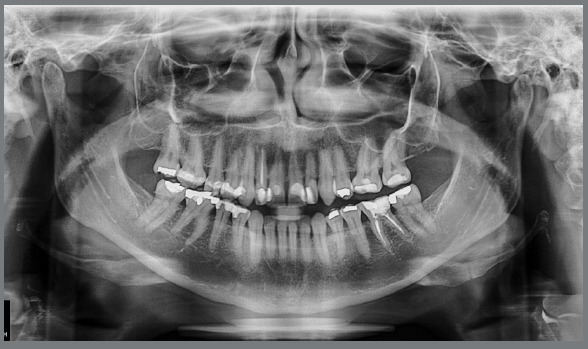
Baseline panoramic radiograph.

**Figure 4:**
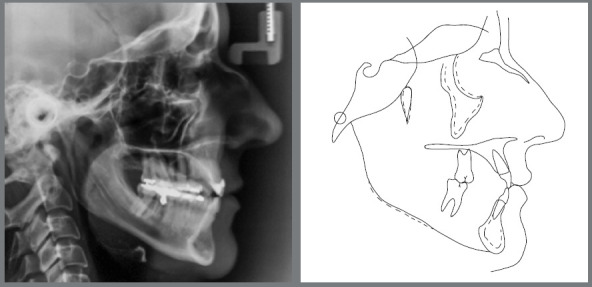
Baseline cephalometric radiograph and cephalometric tracing.

A panoramic radiograph revealed that teeth #25, #18, #28, #48, #38 were missing and that the crown of tooth #26 was inclined mesially. There was generalized horizontal bone loss, but no active periodontal disease (Fig 3). Tooth #36 had an unsatisfactory endodontic treatment, with a partially obturated canal. The cephalometric radiograph (Fig 4) revealed that the maxilla and the mandible were well positioned in relation to the anterior cranial base. The patient had a balanced mesofacial growth pattern. Her maxillary incisors were slightly retruded and had a decreased axial inclination. Her mandibular incisors were slightly protruded, and their axial inclination was satisfactory. Her bone profile was straight, and her soft tissue profile was concave (Table 2).

**Table 2:** Baseline and final cephalometric landmarks.

	MEASURES		Normal	A	B	Difference A/B
Skeletal pattern	SNA	(Steiner)	82°	81°	80°	-1
	SNB	(Steiner)	80°	79°	79°	0
	ANB	(Steiner)	2°	2°	1°	-1
	Wits	(Jacobson)	♀ 0 ±2mm ♂ 1 ±2mm	1.5mm	1mm	-0.5
	Angle of convexity	(Downs)	0°	2°	-2°	-4
	Y-Axis	(Downs)	59°	62°	60°	-2
	Facial Angle	(Downs)	87°	84°	85°	1
	SN.GoGn	(Steiner)	32°	35°	31°	-4
	FMA	(Tweed)	25°	30°	29°	-1
Dental pattern	IMPA	(Tweed)	90°	91°	94°	3
	1.NA (degrees)	(Steiner)	22°	19°	28°	9
	1-NA (mm)	(Steiner)	4 mm	3.5mm	6mm	2.5
	1.NB (degrees)	(Steiner)	25°	24°	25°	1
	1-NB (mm)	(Steiner)	4mm	4.5mm	5mm	0.5
	- Interincisal angle	(Downs)	130°	135°	125°	-10
	1 - APg	(Ricketts)	1mm	2mm	2.5mm	0.5
Profile	Upper Lip - Line S	(Steiner)	0mm	-3mm	-4.5mm	-1.5
	Lower Lip - Line S	(Steiner)	0mm	-4mm	-3mm	1

### TREATMENT OBJECTIVES

The main treatment objectives were: 1) preserve dental aesthetics; 2) correct unilateral SPCB; 3) correct overbite and overjet; and 4) achieve functional occlusion, adequate disclusion and bilateral, simultaneous occlusal contacts.

### TREATMENT OPTIONS

Three treatment options were considered:

1) Left SSRO for constriction and consequent correction of mandibular asymmetry and unilateral SPCB.
2) Extraction of tooth #35, anchorage loss in teeth #36 and #37, and constriction of the left mandibular dental arch.
3) Surgical expansion of the left side of the maxilla, to accentuate the discrete asymmetry, as well as to correct unilateral SPCB and achieve asymmetric arch coordination.

All treatment options would be associated with corrective orthodontic treatment, to restore normal occlusion at the end of the treatment.

Option 1 was undoubtedly the most adequate, because it would act directly on the resolution of bone asymmetry in the mandible, and would correct facial asymmetry. However, the patient refused this option, because she did not want to undergo an invasive and traumatic surgery. She also said she was happy with her dental aesthetics and that asymmetry did not affect her self-esteem. She also refused option 2 because of the need to extract one more tooth (#35), as she already had five missing teeth. Therefore, she chose option 3. The patient received the information that her mandible and face would remain asymmetric, and that the maxillary arch would be more expanded in the left side because of the correction of the unilateral SPCB. 

### SURGICAL ORTHODONTIC TREATMENT AND ORTHODONTIC MECHANICS

#### Maxillary arch

**Figure 5:**
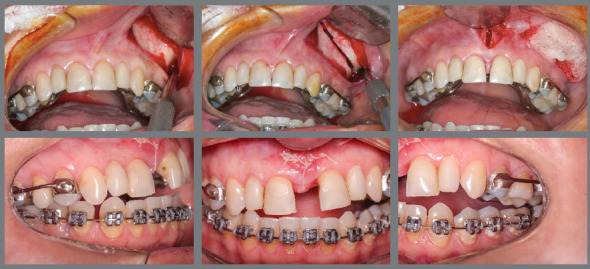
Unilateral Le Fort I osteotomy and unilateral expansion immediately after activations.

After the placement of bands on teeth #14, #24, #16 and #26, impressions of the maxillary arch were taken, and the bands were transferred. A Hyrax palatal expander was fabricated, and the patient was referred to surgery. The procedure consisted of a Le Fort I maxillary segment osteotomy on the left side, from the pyriform aperture to the zygomatic buttress, and midline splitting in the anterior maxilla (Fig. 5A, B, C). An osteotome was used for midline splitting, and the expander screw was activated 8/4 of a turn, to a total of 2 mm. After that, the screw was turned back 4/4 of a turn, to a total of 1 mm, which resulted in a 1-mm diastema between maxillary central incisors. Seven days after surgery, the patient received instructions to activate the screw 2/4 of a turn in the morning and 2/4 in the evening. Weekly return visits were scheduled. Expansion was discontinued when unilateral SPCB was overcorrected, that is, when the palatal cusps of maxillary molars and premolars occluded with the buccal cusps of mandibular molars (Fig. 5D, E, F). During that same visit, the screw was locked in position using self-curing acrylic resin. Occlusal radiographs were taken before the procedure, when the screw was locked in position and before the expander was removed.

The expander was used for retention for six months and then removed. After that, the orthodontic appliance was placed in the maxillary arch. For leveling and alignment, 0.014-in to 0.020-in stainless steel archwires were used to preserve left dental arch asymmetry, as the left side was expanded. Space mesial and distal to teeth #12 and #22 was preserved for later aesthetic reconstruction. Intermaxillary elastics were used to correct the maxillary midline and anchorage loss. A 0.019 x 0.025-in stainless steel archwire was used to complete the treatment and adjust intercuspation. The asymmetry in the maxillary arch was preserved, and torque and bends were used to stabilize the transversal relationship. A panoramic radiograph was requested at the time the last archwire was used, to evaluate root parallelism and to plan future retention. 

#### Mandibular arch

After the brackets and tubes were bonded in the mandibular dental arch, the interproximal reduction of teeth #33, #32, #31, #41, #42 and #43 was used for the correction of anterior crowding and the deviation of the mandibular midline to the left. Leveling and alignment were performed using 0.014-in to 0.020-in stainless steel archwires, and the baseline asymmetry of the mandibular arch was preserved. A 0.019 x 0.025-in stainless steel archwire was coordinated with the maxillary archwire for treatment completion. Completion bends were included to improve intercuspation. 

Occlusion function and arch stability were followed up for 60 days before the appliance was removed. After debonding, a wraparound retainer was prescribed for continuous use for two years, together with a thin 3x3 lingual arch. The patient was seen at each 30 days in the beginning, and after 3, 6, 9 and 12 months. 

## RESULTS

**Figure 6:**
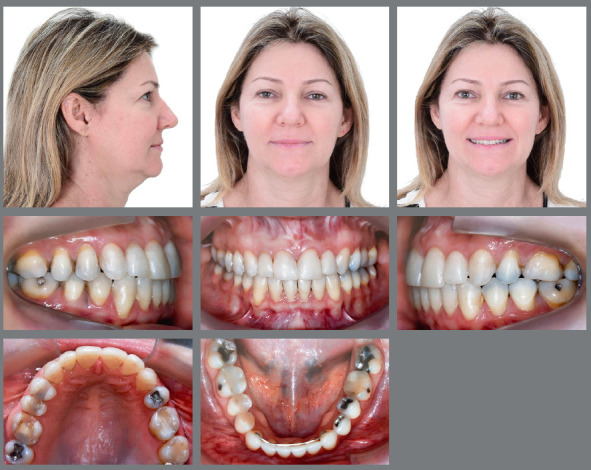
Final facial and intraoral photographs.

**Figure 7:**
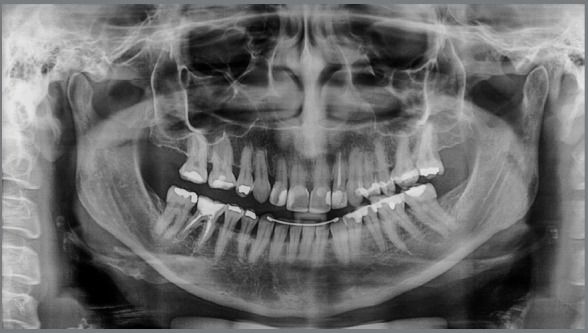
Final panoramic radiograph.

**Figure 8:**
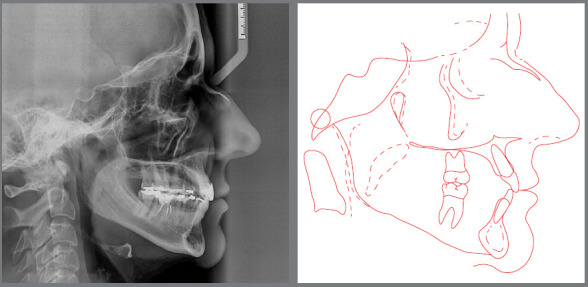
Final cephalometric radiograph and cephalometric tracing.

**Figure 9:**
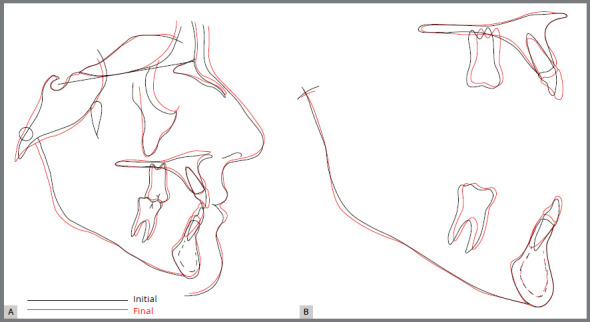
Total (**A**) and partial (**B**) baseline (black) and final (red) cephalometric tracing superimpositions.

The initial objectives of the orthodontic treatment were achieved. Extraoral photographs at the end of the treatment show a harmonious facial profile and smile, at the same time that a slight mandibular asymmetry was preserved in the left side (Fig. 6). Angle Class II, division 2, subdivision left relationship was preserved, and unilateral SPCB was corrected, which restored normal transverse occlusion in the left side. Maxillary and mandibular midlines were coincident with the facial midline, and overbite and overjet were within normal parameters. The slight anteroinferior crowding was corrected. Maxillary lateral incisors received aesthetic restorations to correct their mesiodistal diameter (Fig 6). At the end of the treatment, root parallelism was satisfactory (Fig 7). There were no significant cephalometric changes (Figs 8, 9 and Tab. 2). Her facial profile was preserved: the maxillary incisors were proclined and extruded. 

**Figure 10:**
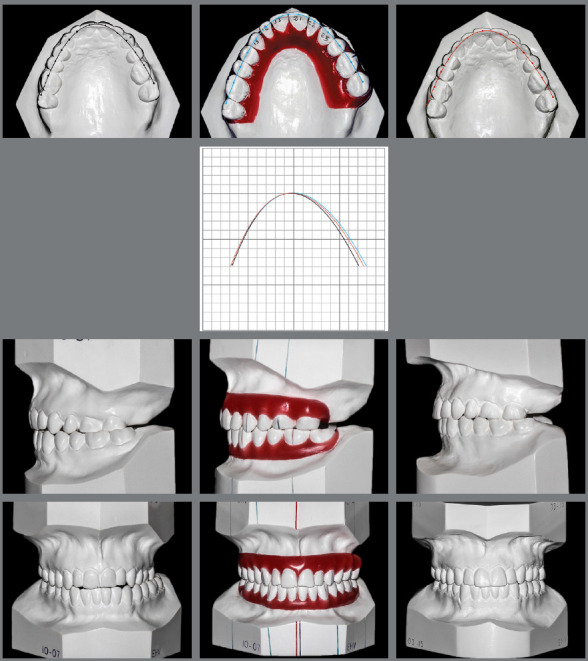
Photographs comparing baseline working casts, orthodontic setup, and final casts; and simulated arch superimpositions on millimeter paper.

## DISCUSSION

SPCB in adults and adolescents whose skeletal maturation is advanced is a challenge, and a corrective surgery is often necessary. The dentoalveolar and skeletal characteristics involved in the several different clinical situations possible should be identified before a decision is made about which approach to use.^4^ ^,^ ^22^ ^-^ ^24^ SPCB correction in skeletally mature patients using conventional RME or dental expansion may lead to unsatisfactory results, with damage to supporting tissues and instability. Therefore, other expansion procedures should be used.^9^ ^,^ ^10^ ^,^ ^12^ ^,^ ^15^ Among those most often used, SARME and MARPE have had good results.^10^ ^,^ ^11^ MARPE was not used in the treatment of this clinical case despite its advantages. It is less invasive and less expensive, its expander is easier to place, and it may be used for the parallel separation of the midpalatal suture. However, it was not an accessible option at the beginning of the treatment. In addition, clinical experience indicates that the use of MARPE is substantially effective in young adults aged 18 to 25 years; however, it has a certain rate of failure for older individuals, such as the patient in this clinical report.

To restore symmetry, many orthodontists prefer to correct the asymmetry at its place of origin because of a cause and effect relationship. If they had to work with this case, they would restore symmetry in the mandible, which was the specific place of origin. To do that, they would perform SSRO in a hospital under general anesthesia. SSRO has some surgical risk, because the dentoalveolar segment is separated from the basal bone of the mandible and repositioned lingually. This procedure requires an extensive surgical intervention and has significant risks, such as segment necrosis, loss of pulp vitality and temporary or permanent paresthesia in the area of the mental nerve. When compared with the surgical risks of conventional orthognathic surgery, SSRO morbidity is higher. Therefore, this surgical approach is not often used.^21^ ^-^ ^24^ SSRO may result in a greater constriction in the canine region than in the molar region,^20^ which would be unfavorable in this case, because constriction was more necessary in the region of tooth #36, with an 8-mm expansion, in relation to tooth #46. The patient refused this option because of the complexity of the surgical procedure in the mandible. Therefore, after considering the specific characteristics of the case and preparing the orthodontic setup, we chose to accentuate left maxillary asymmetry using SARME to correct SPCB. The procedure was performed in the office, and there was no need of hospitalization or general anesthesia. It should be stressed that SARME also poses risks to patients; however, these risks are less significant than those posed by SSRO, as discussed above. Glassman et al.^16^ found that no unilateral osteotomies in their study were performed to camouflage another asymmetry, which indicates that the clinical case described in this report received a different treatment for unilateral SPCB. 

In cases of unilateral SPCB, the expansion can be uni or unilateral. In case of bilateral expansion, osteotomy should include all the maxilla. In contrast, when the condition affects only one side, surgery is performed only in that side. The case reported here illustrates this SARME modality, as the osteotomy was performed only in the left side and promoted the asymmetric expansion of the maxilla. This procedure also opened the diastema between the central incisors, which increased the space available in the maxillary dental arch. As the osteotomy was not performed on the side without a crossbite, there was no significant expansion in this segment.^16^ ^,^ ^17^

Before the orthodontic treatment in this clinical report, the patient had a Class II, division 2, subdivision left relationship because of loss of tooth #25 and the consequent mesial movement of #26. At the end of the treatment, the Angle Class II molar relationship was preserved in the left side, and there was a correct Class I occlusion of the canines in both sides. This is in agreement with the consensus that this molar anteroposterior relationship is stable and functional.^25^ ^-^ ^28^ During corrective orthodontic treatment, one of the objectives was the improvement of left side intercuspation, to make it functional. To promote an adequate occlusal contact with the mandibular arch, molars in Class II relationship should not be offset.

Another treatment option presented to the patient included the extraction of tooth #35 and the mesial movement of teeth #36 and #37, which would be moved to a narrower area of the mandibular arch, correcting SPCB and resulting in a Class I molar relationship. The anteroposterior relationship of canines and molars in both sides was already satisfactory at the beginning of the treatment and should be preserved. Although plausible, this treatment option may lead to problems, such as a probable uprighting of mandibular incisors, which would worsen her facial profile and substantially increase her slight sagittal mandibular dental asymmetry. Moreover, mandibular bone asymmetry, which had a skeletal origin, would persist even though her unilateral SPCB was corrected.

After the correction of unilateral SPCB, orthodontic treatment became easier, because the patient has a minor tooth-size/arch-length discrepancy (-2 mm), a normal curve of Spee, and well-positioned mandibular incisors (1.NA=24^o^). Treatment plan included the interproximal reduction of mandibular incisors to treat crowding and correct the mandibular midline. Intermaxillary elastics and torque control were used to adjust occlusion and intercuspation, as well as to achieve the proclination of maxillary incisors. These orthodontic mechanics ensured the correction of overbite and overjet, as well as the improvement of her facial profile. 

Facial asymmetries, usually visible when larger than 4 mm, are not well accepted by patients. Asymmetries are among the most difficult problems to treat and are often corrected only by means of surgical procedures.^1^ ^,^ ^2^ However, the patient in this case had a slight facial asymmetry noted only by the people closest to her and by specialized professionals. The treatment option selected for this case did not aim at the correction of that asymmetry, as the patient was comfortable with this condition. 

The results simulated in the orthodontic setup were very close to the actual clinical results, which confirms the great value of this physical or virtual diagnostic modality in complex cases (Fig 10). This case also illustrates that, although asymmetry is a very important aesthetic and functional problem, the correct and thorough coordination of dental arches is essential to achieve a good treatment completion.

## CONCLUSION

Asymmetries are usually corrected at the site where they originate. This report described a clinical case in which asymmetry was approached differently, as the symmetric basal bone was surgically expanded asymmetrically, thus correcting unilateral SPCB. At the end of the treatment, correct occlusion and satisfactory facial aesthetics were achieved by means of a combination of surgical and orthodontic treatments.
